# Initiation of Hemodialysis After Eight Years Following the Diagnosis of Stage 5 Chronic Kidney Disease: A Case Report

**DOI:** 10.7759/cureus.11357

**Published:** 2020-11-06

**Authors:** Mohamed R Almajed, Salman J Ali Jan

**Affiliations:** 1 Medicine, Salmaniya Medical Complex, Manama, BHR; 2 Medicine, Royal College of Surgeons in Ireland - Medical University of Bahrain, Busaiteen, BHR; 3 Medicine, King Hamad University Hospital, Muharraq, BHR; 4 Medicine, Royal College of Surgeons in Ireland, Dublin, IRL

**Keywords:** chronic kidney disease, dialysis, renal replacement therapy, end stage renal disease, timing of dialysis initiation

## Abstract

With the growing global rates of diabetes and hypertension, chronic kidney disease (CKD) appears to be a major contributor to morbidity and all-cause mortality. In recent years, there has been growing controversy regarding the optimal timing for the initiation of hemodialysis in this patient cohort. In this report, we present the case of a 52-year-old female with a 15-year history of CKD who was admitted to the hospital with clinical manifestations of uremia, volume overload, and symptomatic anemia. The patient presented with fatigue, nausea, progressive shortness of breath, and lightheadedness for two weeks, which had limited the activities of daily living. For the past eight years, her estimated glomerular filtration rate (GFR) had ranged from 5 to 15 mL/min/1.73 m^2^, consistent with kidney failure seen in stage 5 CKD. Prior to her recent admission, the patient had been grossly asymptomatic and had been responsive to medical therapy. After appropriate management with hemodialysis, a transfusion of packed red blood cells, and medication adjustment, the patient was scheduled for maintenance dialysis through an arteriovenous fistula. She had no further complaints and her laboratory abnormalities were found normalized at the six-month follow-up. This case report presents the survival and outcome of a patient with stage 5 CKD, who was only initiated on hemodialysis eight years after her diagnosis.

## Introduction

Chronic kidney disease (CKD) is a long-term decrease affecting kidney function, defined as a condition where the glomerular filtration rate (GFR) drops to a level of <60 mL/min/1.73 m^2^ for a period of more than three months [1]. The global prevalence of CKD is 13.4%, a rate that has been rising due to an increase in the prevalence of diabetes mellitus and hypertension, its two most common causes [2-3]. Despite the significant morbidity and mortality associated with CKD, it has been a largely overlooked public health issue with a high disease burden that is not mitigated by the limited resources allocated to it [4].

Although reversible, most cases of CKD progress to kidney failure and require renal replacement therapy, of which dialysis is the most common modality. The timing of dialysis initiation has been a matter of dispute for years, with different guidelines recommending different criteria and indications. Conventionally, the Initiating Dialysis Early and Late (IDEAL) Trial found that early initiation of dialysis did not have a beneficial effect on patient survival or quality of life [5]. Recent studies have favored the assessment of a patient's symptoms and holistic clinical picture and the use of a strict estimated GFR (eGFR) level in guiding the decision to initiate dialysis [6-7].

In this report, we present the case of a patient with kidney failure secondary to CKD who was initiated on hemodialysis eight years after her diagnosis.

## Case presentation

A 52-year-old Caucasian female with a significant 15-year history of CKD secondary to hypertension was admitted to hospital with chief complaints of fatigue, nausea, progressive activity-limiting shortness of breath, and lightheadedness for two weeks. These symptoms were more severe than her previous episodes and were associated with a two-month history of worsening lower limb swelling and pruritus. The patient demonstrated signs consistent with uremia, volume overload, and symptomatic anemia. The patient’s vital signs were remarkable for tachycardia and blood pressure of 156/92 mmHg despite being within the target blood pressure of <130/80 mmHg since her diagnosis. On clinical examination, she had generalized muscle wasting, conjunctival pallor, bilaterally reduced breath sounds and dullness to percussion at the lung bases, and bilateral lower limb 3+ pitting edema.

For the past eight years, her eGFR had ranged from 5 to 15 mL/min/1.73 m^2^, consistent with kidney failure seen in stage 5 CKD. The patient’s past medical history was remarkable for hypertension, but her family history was unremarkable. She had been consulting regularly with her nephrologist and had undergone kidney function tests every three months. Laboratory results within the past six months had indicated a further decline in kidney function to an eGFR level of 4 mL/min/1.73 m^2^ and persistently low hemoglobin levels ranging from 8 to 9 g/dL. Prior to her admission, the patient had been generally asymptomatic. She had been responsive to medical therapy consisting of ramipril 5 mg orally twice daily, furosemide 40 mg orally twice daily, darbepoetin alfa 60 mcg subcutaneous once every two weeks, and calcium carbonate 1,250 mg orally three times a day. Her regimen was consistent with the Kidney Disease Improving Global Outcomes (KDIGO) guidelines and she had no previous hospitalizations due to CKD. Her regimen had remained the same throughout her care plan. The patient had been compliant with appropriate dietary advice, which included fluid restriction, phosphate restriction, and a low-sodium diet. She had maintained an active lifestyle by participating in aerobic exercise classes several times a week. The patient had remained consistently hesitant to consider renal replacement therapy due to her fear of complications and the potential inconveniences incurred.

Investigations revealed a deterioration in kidney function from the patient’s baseline; she had a creatinine of 1,047 μmol/L, eGFR of 3 mL/min/1.73 m^2^, and urea of 47.5 mmol/L. Blood tests showed normocytic normochromic anemia with a hemoglobin level of 6.8 g/dL. Acute blood loss and iron deficiency were ruled out. She also had a high anion-gap metabolic acidosis with hyperkalemia of 5.9 mmol/L, hyperphosphatemia, and hypocalcemia. Chest X-ray (CXR) was remarkable for a mild bilateral pleural effusion of the lower lung zones consistent with volume overload. Other investigations including coagulation profile, cardiac biomarkers, urinalysis, and electrocardiogram (ECG) were unremarkable.

The patient was managed in a high-dependency unit. She met the indication for dialysis and hemodialysis was commenced acutely via a central catheter. She received a transfusion of packed red blood cells, and appropriate medication dose adjustments were made. On discharge, the patient’s laboratory abnormalities and pleural effusion had resolved. She was scheduled for maintenance dialysis and referred for arteriovenous fistula insertion.

On follow-up six months later, the patient was receiving dialysis three times weekly through an arteriovenous fistula. She had no further symptoms or complications.

## Discussion

Kidney failure secondary to CKD is characterized by uremia due to the critical loss of kidney function to a point that is unsustainable for life. Although this state commonly occurs in the vast majority of patients at a GFR level of <15 mL/min/1.73 m^2^, some patients may be able to withstand such levels without the development of uremic symptoms whereas others develop overt failure at higher GFR levels [1].

Renal replacement therapy is a life-sustaining intervention for patients who develop CKD with kidney failure; hemodialysis and peritoneal dialysis are the most common modalities. However, the timing of dialysis initiation is a rather disputed topic. The European Renal Best Practice Advisory Board strongly recommends that initiation is considered in those with presentations of uremia, inability to control hydration status or blood pressure and/or malnutrition, and an eGFR of <15 mL/min/1.73 m^2^ [8]. The Canadian Society of Nephrology, however, advises initiating dialysis with the first onset of a clinical indication, such as fluid overload or refractory hyperkalemia, or with a decline in the eGFR to 6 mL/min/1.73 m^2^ or lower [9]. Following the latter guidelines, proof of laboratory results within the past six months would qualify our patient for dialysis on the basis of her eGFR alone.

**Figure 1:**
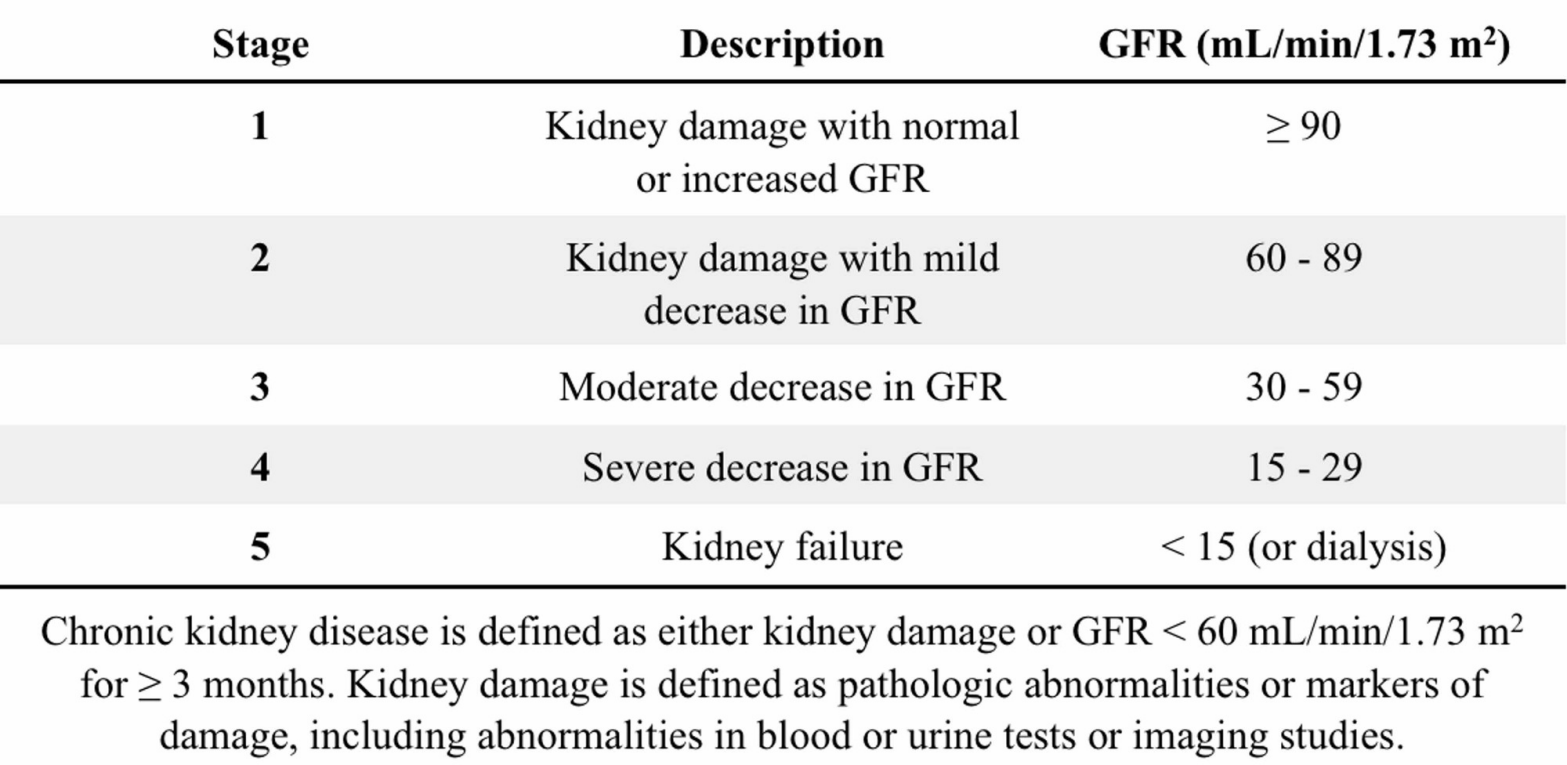
KDOQI stages of chronic kidney disease KDOQI: Kidney Disease Outcomes Quality Initiative; GFR: glomerular filtration rate

Dialysis initiation is further complicated by the confusion caused by the Kidney Disease Outcomes Quality Initiative (KDOQI) classification of CKD. This classification stratifies patients into five categories of decreased kidney function and is the most globally accepted classification in clinical practice (Figure 1). Despite providing a simple universal method of communicating a patient’s condition, the classification imposes a linear definition on a nonlinear disease. For instance, the difference in kidney functions between categories G3a and G5 has little clinical relevance as they can all be medically managed until the development of overt kidney failure [10]. Thus, a need to consider assessment tools other than eGFR, such as the assessment of symptom prevalence, the severity of complications, and patient education, are potential areas of research in the future [11].

Furthermore, as it is the final category, stage G5 has been become erroneously synonymous with end-stage renal disease (ESRD) among the general medical community with an implication that these patients all require dialysis [12]. ESRD is defined as the occurrence of kidney failure that necessitates renal replacement therapy, irrespective of the GFR level [1]. Clinical features suggestive of such a state include uremic serositis, acid-base and electrolyte abnormalities, and pruritus; inability to control volume status or blood pressure; a progressive deterioration in nutritional status refractory to dietary intervention; and cognitive impairment [5]. This presentation typically occurs three to four years after the GFR falls below 15 mL/min/1.73 m^2^, to levels between 6 and 9 mL/min/1.73 m^2^. This is based on an average GFR decline of 2.65 mL/min/1.73 m^2^,^ ^which is seen in patients with GFR categories G3a and G5 [5,13].

It has been observed that CKD patients with a GFR below 15 mL/min/1.73 m^2^ have a poor survival rate. A study in such patients managed medically determined their survival rate to be 68% at one year, 47% at two years, and 20% at five years [14]. Another study found that the median survival from entry into stage 5 CKD in patients managed conservatively was 21.2 months [15].

In our case, the patient had maintained adequate kidney function at a GFR below 15 mL/min/1.73 m^2^ for eight years with minimal symptoms before progressing to ESRD, offering a greater role for lifestyle modifications and medical therapy. The patient was followed up and no further deterioration was reported after the initiation of hemodialysis.

Evidently, patient education on diet, exercise, and medication correlates with a significant delay in the progression of CKD [16]. This is particularly important in those who choose to defer dialysis. Recent data recommend a diet containing 0.6 g/kg per day of protein for those with a GFR between 13 and 25 ml/min/1.73 m^2^, which was roughly consistent with the nutritional guidance followed by our patient [17]. Administration of oral sodium bicarbonate, angiotensin-converting enzyme inhibitors, and angiotensin receptor blockers can provide similar benefits [18]. It would also be worthwhile for future research to expand on the role of exercise regimen and dietary advice on CKD progression.

## Conclusions

The rapidly successive progression of CKD with a GFR level of <15 mL/min/1.73 m^2^ to ESRD is common and has pushed physicians to initiate dialysis early in anticipation of decompensation. In rare cases, patients such as the one in our case are able to survive for several years on medical management with minimal symptoms of kidney failure. It was the combined success of optimal medical therapy, adherence to follow-up appointments, and, most importantly, dietary control that led to this outcome. This report highlights the importance of adopting a patient-centered approach in the decision-making process relating to dialysis initiation and deferring initiation in asymptomatic individuals. Further research is required in this field to arrive at firm conclusions about this topic.
